# Antibacterial and antibiofilm activities of iodinated hydrocarbons against *Vibrio parahaemolyticus* and *Staphylococcus aureus*

**DOI:** 10.1038/s41598-024-55479-7

**Published:** 2024-04-22

**Authors:** Oluwatosin Oluwaseun Faleye, Olajide Sunday Faleye, Jin-Hyung Lee, Jintae Lee

**Affiliations:** School of Chemical Engineering, Yeungnam University, 280 Daehak-Ro, Gyeongsan, 38541 Republic of Korea

**Keywords:** Biofilms, Antibiotics

## Abstract

Food-related illnesses have become a growing public concern due to their considerable socioeconomic and medical impacts. *Vibrio parahaemolyticus* and *Staphylococcus aureus* have been implicated as causative organisms of food-related infections and poisoning, and both can form biofilms which confer antibiotic resistance. Hence, the need for continuous search for compounds with antibiofilm and antivirulence properties. In this study, 22 iodinated hydrocarbons were screened for their antibiofilm activity, and of these, iodopropynyl butylcarbamate (IPBC) was found to effectively control biofilm formation of both pathogens with a MIC of 50 µg/mL which was bactericidal to *V. parahaemolyticus* and *S. aureus*. Microscopic studies confirmed IPBC inhibits biofilm formation of both bacteria and also disrupted their mixed biofilm formation. Furthermore, IPBC suppressed virulence activities such as motility and hemolytic activity of *V. parahaemolyticus* and the cell surface hydrophobicity of *S. aureus*. It exhibited a preservative potential against both pathogens in a shrimp model. IPBC disrupted the cell membrane of *S. aureus* and *V. parahaemolyticus* and differentially affected gene expressions related to biofilm formation and virulence. Additionally, it displayed broad-spectrum antibiofilm activities against other clinically relevant pathogens. These findings indicate IPBC offers a potential means of controlling infections mediated by *Vibrio* and *Staphylococcus* biofilms.

## Introduction

Foodborne diseases caused by microorganisms have recently emerged as a substantial global public health issue in both developed and developing nations due to their high morbidity and mortality rates (WHO, 2015). Furthermore, more than 80% of human acute and reoccurring bacterial infections and over 60% of foodborne outbreaks are associated with biofilm formation^1^.

Microbial biofilms are communities of microorganisms attached irreversibly to surfaces and enclosed in a matrix of extracellular polymeric substances comprised of biomolecules such as eDNA, polysaccharides, and proteins that serve as a barrier to disinfectants and antibiotics^1^. Biofilm formation in the food industry is a major near uncontrollable problem due to the abundance of nutrients and surfaces suitable for microbial attachment and the complexity of processing equipment. In addition, the contamination in this industry is prevalently caused by mixed-species biofilms rather than the single species and this phenomenon has complicated their control, treatment or elimination from food processing plants and equipment^2,3^.

*Vibrio parahaemolyticus* is a rod-shaped, Gram-negative, flagellated, halophilic, motile bacterium found in marine and coastal environments and is a major contaminant of seafoods. Biofilms produced by *V. parahaemolyticus* have been reported on oysters, clams, fish, shrimps, mussels, scallops, and periwinkles^4,5^. This bacterium is also capable of causing acute gastroenteritis resulting from the ingestion of contaminated raw or undercooked or improperly handled marine food products^5^. The high prevalence and frequency of outbreaks caused by this pathogen are alarming, especially in the USA and Asia^6^. Furthermore, *V. parahaemolyticus* possesses a number of virulence factors, including polar and lateral flagella, and adhesion factors that increase its ability to invade hosts, attach to surfaces, and produce biofilms^6^. *V. parahaemolyticus* in biofilm state is capable of enhanced virulence^7^ and its transcriptional protein ToxR directly regulates both biofilm formation and thermostable direct hemolysin^4,8^.

*Staphylococcus aureus* is a non-motile, Gram-positive coccus bacterium that exists singly or in paired cocci that resemble grape clusters^9^. *S. aureus* is a normal component of body flora and is found in hair, skin, and nasal cavities, and thus, due to poor hygiene and improper handling easily contaminates foods and food processing equipment^10^. *S. aureus* is also known to produce staphylococcal enterotoxins responsible for food poisoning with symptoms including dry throat, nausea, vomiting, diarrhea, headache, and double vision. Furthermore, *S. aureus* biofilms can confer antibiotic resistance to chronic infections such as endocarditis and osteomyelitis which could be life threatening^11,12^. Also, it was reported in wound or surgical site infections and as opportunistic pathogen in COVID-19 related cases^13,14^. *V. parahaemolyticus* and *S. aureus* biofilms have also been detected on food-contacted surfaces and the consumption of food contaminated by them has been frequently linked to food-borne diseases^10,15,16^. Consequently, there is a need for potent bioactive compounds that can control *V. parahaemolyticus* and *S. aureus* biofilms and their pathogenic factors.

Halogenated hydrocarbons (halocarbons) are hydrocarbon compounds in which at least one hydrogen atom is replaced by a halogen^17^, and they have proven to be very useful for controlling microorganisms^18,19^. In our previous studies, we established that straight chain fatty acids as well as halogenated derivatives of natural compounds including iodinated derivatives possessed antibiofilm potentials^20–24^. Halogenation has been employed to improve intracellular delivery, metabolic stability, enhance target binding affinity, and potentiate antimicrobial activities^25,26^. Therefore, we hypothesized that halogenated hydrocarbons may be viable alternative for biofilm control. Hence, in this study, 22 selected iodinated compounds were evaluated for their antibiofilm activity against *S. aureus* and *V. parahaemolyticus* using a crystal violet assay*.* Thereafter, potent compounds with excellent activity were further dose dependently examined against the planktonic and biofilm cells and their effects on virulence factors, viz. swimming and swarming motilities, cell surface hydrophobicity, hemolysin and staphyloxanthin production. The effects of iodopropynyl butylcarbamate (IPBC) on dual species biofilm formation involving *S. aureus* and *V. parahaemolyticus* were examined by live imaging and scanning electron microscopy (SEM). Similarly, broad-spectrum antibiofilm activities against other pathogens such as *Candida albicans*, uropathogenic *Escherichia coli,* and *Vibrio harveyi* were evaluated in addition to its preservative capacity in food system. Finally, the possible mechanism responsible for the antimicrobial activities against *V. parahaemolyticus* and *S. aureus* and its effects on biofilm- and virulence-related gene expressions were investigated.

## Materials and methods

### Bacterial strains, growth conditions, and chemicals

Methicillin-sensitive *S. aureus* ATCC 6538, *V. parahaemolyticus* ATCC 17802, *Vibrio harveyi* ATCC 14126, uropathogenic *Escherichia coli* O6:H1 strain CFT073 ATCC 700928, and *Candida albicans* DAY 185 (American Type Culture Collection, Manassas, VA, USA) were used in this study. *V. parahaemolyticus* was cultured in Luria–Bertani broth (LB) supplemented with 3% (w/v) sodium chloride (marine Luria–Bertani broth—mLB) at 30 °C, while *S. aureus* was cultured in Luria–Bertani broth (LB) at 37 °C. *S. aureus* and *V. parahaemolyticus* were streaked from − 80 °C glycerol stocks on Luria–Bertani and marine Luria–Bertani plates, respectively, and single colonies from plates were cultured in LB or mLB overnight (representing a growth period of 12–13 h with bacteria grown to stationary phase) at 250 rpm at 37 °C and 30 °C respectively. Assay results were the averages of at least two independent cultures conducted in triplicate.

Twenty-two iodinated hydrocarbons, namely, 1-chloro-4-iodobutane, 1-chloro-6-iodohexane, chloroiodomethane, 1-chloro-3-iodopropane, 1,4-diiodobutane, 1,10-diiododecane, 1,2-diiodoethane, 1,6-diiodohexane, diiodomethane, 1,5-diiodopentane, 1,3-diiodopropane, 1-iodododecane, iodoethane, iodoform, 1-iodoheptane, 1-iodohexane, 1-iodononane, 1-iodooctane, 1-iodopropane, 2-iodopropane, 3-iodopropanol and iodopropynyl butylcarbamate (IPBC) were evaluated and they were purchased from Combi-Blocks Inc. (San Diego, California, USA). Stock solutions were prepared in DMSO (dimethyl sulfoxide) at 100 mg/mL and stored at − 20 °C until required. The concentration of DMSO did not exceed 0.1% v/v in experiments with no effect on biofilm formation or cell growth.

### Initial screening and dose-dependent antibiofilm activities

The antibiofilm activities of the 22 iodinated hydrocarbons were initially evaluated against *S. aureus* and *V. parahaemolyticus*, as reported^21^. Overnight cultures of *S. aureus* and *V. parahaemolyticus* were reinoculated at 1:100 in LB or mLB broth, respectively, with or without each iodinated hydrocarbon at 100 μg/mL. Samples (300 μL) were then transferred to 96-well polystyrene plates (SPL Life Sciences, Korea) and incubated under static conditions for 24 h at 37 °C or 30 °C, respectively. Planktonic cell growth (OD_620_) was measured with Multiskan EX microplate reader (Thermo Fisher Scientific, Waltham, MA, USA). For biofilm quantification, media containing planktonic cells were discarded from wells by gently immersing plates three times in distilled water (dH_2_O). Biofilm cells were then stained with 0.1% crystal violet (CV) for 20 min, rinsed plates with dH_2_O, and CV was dissolved with 95% ethanol. Absorbances were measured at 570 nm with a Multiskan EX microplate reader. The antibiofilm activities of active derivatives were then further examined using the same method at 0, 5, 10, 20, 50, or 100 µg/mL.

### Biofilm dispersal assay

The ability of IPBC to disperse preformed biofilms was also examined. *S. aureus* and *V. parahaemolyticus* were diluted in their respective media and inoculated in 96-well plates without IPBC for 24 h at 37 °C or 30 °C, respectively. Broths containing planktonic cells were then removed by pipetting, and plates were washed with phosphate-buffered saline (PBS, pH 7.4) to remove non-attached cells. Different doses of IPBC in LB and mLB were added to wells and incubated for another 24 h. CV staining was carried out as stipulated in “Initial screening and dose-dependent antibiofilm activities”, followed by absorbance measurement at OD_570_.

### Cell growth assessments

The growths of *S. aureus* and *V. parahaemolyticus* planktonic cells treated with different concentrations of the active hydrocarbons were monitored as previously described^27^. Overnight cultures of bacteria were diluted (1:100) in appropriate media, and treated with or without IPBC at 0, 10, 20, 50, or 100 µg/mL. Thereafter, 300 µL aliquots were dispensed into 96-well plates and incubated at 30 °C and 37 °C for *V. parahaemolyticus* and *S. aureus,* respectively, under static conditions. Bacterial growths were assessed by measuring absorbances at 600 nm two hourly for 24 h.

### Time-to-kill assays

The bactericidal and bacteriostatic effects of IPBC on *V. parahaemolyticus* and *S*. *aureus* were investigated as previously described^28^. Overnight cultures of *V. parahaemolyticus* or *S*. *aureus* were reinoculated at 1:100 dilution with or without IPBC at MIC or 2 × MIC and were incubated at 30 °C or 37 °C respectively with shaking (250 rpm). Samples (100 µL) were taken 4 hourly, serially diluted, plated on appropriate agar plates, and incubated at 30 °C or 37 °C for V*. parahaemolyticus* or *S*. *aureus* respectively. Colonies were then counted and calculated as follows:

$${\text{CFU}}/\mathrm{mL }= \frac{number \;of \;colony \;units \times dilution \;factor}{volume \;plated \;(mL)}$$

### Biofilm visualization by live imaging and SEM

To visualize the effects of IPBC on *V. parahaemolyticus* and *S*. *aureus* biofilms and morphologies, 300 μL of cells appropriately diluted (1:100) in the presence of IPBC at 0, 10, 20, 50, or 100 µg/mL were dispensed into 96-well plates and incubated at 30 °C or 37 °C, respectively. Planktonic cells were removed by washing with PBS (pH 7.4) three times, and biofilms were examined using the iRiS Digital Cell Imaging System (Logos BioSystems, Anyang, Korea). Resulting biofilm images were reconstructed as 2D and 3D color-coded pictures using ImageJ (https://imagej.nih.gov/ij/index.html).

The SEM study was performed as previously reported^21^. Briefly, 300 μL of *V. parahaemolyticus* or *S*. *aureus* diluted cells (1:100) in the presence of IPBC at 0, 50, or 100 µg/mL were dispensed into 96-well plates containing a piece of sterile nylon filter membrane (0.4 × 0.4 mm^2^) and incubated without agitation at 30 °C and 37 °C for 24 h. Membranes were gently removed, and attached biofilms were fixed with a 2.5% glutaraldehyde/2% formaldehyde mixture for 24 h and dehydrated using an ethanol series (50–99%). After critical-point drying (HCP-2, Hitachi, Tokyo, Japan) and platinum sputter-coating, films were observed under an S-4800 scanning electron microscope (Hitachi, Tokyo, Japan) at an accelerating voltage of 15 kV and magnifications of × 10,000 to 35,0000.

### Swimming and swarming motility assays

The potential of IPBC to inhibit *Vibrio* motility phenotypes was evaluated, as reported previously^29^. Semi-solid mLB plates containing 0.3% agar and IPBC (0, 10, 20, 50, or 100 μg/mL) were used to determine swimming motility. 1 μL of an overnight culture was inoculated in the center of the agar. To assess swarming motility, mLB plates containing 0.5% agarose and IPBC (0, 10, 20, 50, or 100 μg/mL) were used. Plates were incubated at 30 ℃ for 24 and 48 h. Areas covered by migrating cells were measured and photographed, and plates without IPBC served as controls.

### Hemolysis assay

The ability of IPBC to prevent red blood cell lysis of *V. parahaemolyticus* and *S. aureus* was assessed as previously described with slight modification^21,30^. For *V. parahaemolyticus*, the antihemolytic effects were examined in both solid (Kanagawa phenomenon) and liquid media. The Kanagawa phenomenon was investigated on Wagatsuma blood agar prepared in 1 L distilled H_2_O according to previous report with slight modification^31^. The media was steamed for 30 min (without autoclave) and cooled to 45 °C. Thereafter, the defibrinated sheep red blood cell (MBcell, Seoul, Korea; 50 mL) was added to the medium, swirled and poured into sterile petri dishes with or without IPBC (0, 25, 50 or 100 μg/mL). Plates were thoroughly dried and about 10 μl of overnight culture of *V. parahaemolyticus* grown in mLB was dropped aseptically on the Wagatsuma blood agar and incubated at 37 °C for 36 h. The Kanagawa phenomenon was indicated by a characteristic halo surrounding the growth due to β-hemolysis^32^.

For additional hemolysis assay using a liquid medium, *V. parahaemolyticus* cells cultured overnight were diluted in 2 mL mLB at 1:100 and incubated with IPBC at 0, 10, 20, 30, 40, or 50 μg/mL for 24 h with shaking at 250 rpm. Then, 250 µL of IPBC treated overnight culture of *V. parahaemolyticus* was added to 1 mL of 5% sheep blood erythrocytes obtained by centrifugation (3,000 rpm, 5 min) and washed with PBS (pH 7.4). The mixtures were incubated at 250 rpm for 5 h at 37 °C and the cells were separated by centrifuging at 10,000 rpm for 10 min, and supernatant absorbances were measured at 543 nm^33^. Similar procedures were followed for *S. aureus* except that it was carried out in liquid LB medium with 3.3% sheep red blood cells, 300 µL of treated culture and 1 h incubation period for the hemolysis^21^.

### Cell surface hydrophobicity

The effects of IPBC on the surface hydrophobicities of *S. aureus* and *V*. *parahaemolyticus* were assessed using microbial adhesion to hydrocarbon, as previously reported^34^. *V. parahaemolyticus* and *S. aureus* (1:100 dilution) were cultured in mLB or LB (1 mL) and incubated with or without IPBC at 30 °C or 37 °C, respectively, for 24 h at 250 rpm. After incubation, cells were centrifuged at 10,000 rpm for 15 min at 4 °C, supernatants were discarded, pellets washed twice with PBS (pH 7.4), and further diluted in the same buffer to an absorbance of ~ 0.5 (Ao) at 600 nm. 4 mL of these suspensions was dispensed into glass tubes and 1 mL of toluene and hexadecane were added for *V. parahaemolyticus* and *S. aureus*, respectively. Mixtures were vortexed vigorously (90 s) and left at room temperature for 30 min to settle until the aqueous phase was separated from the organic phase. The optical density (OD) of the aqueous phase was then determined at 600 nm (Ai), and the percentage surface hydrophobicity was expressed as: Hydrophobicity (H) % = (Ao − Ai)/(Ao) × 100.

### Staphyloxanthin production

Briefly, overnight culture of *S. aureus* was diluted in LB medium (~ 10^7^ CFU/mL) and kept for 24 h at 37 °C in the presence or absence of IPBC (0, 10, 20, 30, or 40 μg/mL). Post incubation, 1 mL of the samples was harvested by centrifugation at 10,000 for 10 min, washed with PBS (pH 7.4) and the pellets were optically observed for yellow-golden coloration signifying pigment production and images of the pellets were taken. For quantification, staphyloxanthin was further extracted from the pellet and measured as previously established^21^.

### Antibiofilm activities against other pathogens and dual biofilm formation

The antibiofilm efficacy of IPBC was evaluated against other pathogens such as *V. harveyi* (ATCC 14126) uropathogenic *E. coli* (UPEC) O6:H1 strain CFT073 (ATCC 700928) and *C. albicans* (DAY 185). Briefly, the assay was conducted as detailed in “Initial screening and dose-dependent antibiofilm activities” for all pathogens. Using 1:100 dilutions, *V. harveyi* and UPEC biofilms were formed in marine Luria–Bertani (mLB) and nutrient broth (NB) respectively, while *C. albicans* was assayed in potato dextrose broth (PDB) at a dilution of 1:50. In addition, the ability of IPBC to control mixed *S. aureus*/*V. parahaemolyticus* biofilm formation was investigated by mixing equal volumes of LB and mLB and inoculating the mix with equal volumes of diluted cells (1:100). Then 300 µL of mixed cells treated with or without IPBC were dispensed into 96-well plates and incubated for 24 h at 30 °C without agitation. In both cases, biofilm quantification was carried out as described in “Initial screening and dose-dependent antibiofilm activities”. Diluted *V. parahaemolyticus* or *S. aureus* served as controls. Additionally, the effect of IPBC on mixed films was also investigated by live imaging and SEM, as described in “Biofilm visualization by live imaging and SEM”.

### Preservative potentials of IPBC in a shrimp model

The assay was carried out as previously reported with slight modification^35^. Briefly, beheaded shrimps (*Litopenaeus vannamei*) purchased from a local mart in Gyeongsan, Korea, were thoroughly washed with distilled water and irradiated under ultraviolet (UV) for 25 min to minimize the background flora. The shrimps were cut into pieces (1 g) and inoculated with 1:100 dilution (~ 10^7^ CFU/mL) of *V. parahaemolyticus* and *S. aureus* separately and equal volumes of both pathogens for the dual antibacterial function and kept for 15 min. Thereafter, they were air-dried for 45 min in the biosafety cabinet, grouped and dipped in the various concentrations (0, 25, 50 or 100 µg/mL) of the derivatives for 10 min. The treated shrimps were kept in sterile bags and incubated at 4 °C for 6 days. The shrimp sample was taken at a 2-days interval, homogenized, serially diluted in PBS (pH 7.4) and spread on mLB or LB agar. The colonies were counted and expressed as log CFU/g after 24 h incubation at 30 and 37 °C.

### N-Phenyl-1-naphthylamine (NPN) uptake assay

NPN uptake was employed to determine the impact of IPBC on cell wall integrity of both bacteria, as described by^27^. Bacterial suspensions grown to the exponential phase were centrifuged for 10 min at 4 °C (4000 rpm) and rinsed two times with PBS (pH 7.4). Optical densities (OD_600_) of the bacterial suspensions were then adjusted to 0.5, and cells were treated with IPBC at 0, ½ × MIC, MIC, or 2 × MIC or benzalkonium chloride (the positive control) and incubated at 30 °C for *V. parahaemolyticus* and 37 °C for *S. aureus* for 1 h. NPN solution (15 µL of 10 mM; Sigma-Aldrich, Seoul, Korea) was added to 200 µL of bacterial samples, and fluorescence was measured immediately with a JASCO-F-2700 spectrophotometer (Hitachi, Tokyo, Japan) at an excitation and emission wavelengths of 360 and 460 nm, respectively.

### Quantitative real-time polymerase chain reaction (qRT-PCR)

Cultures of *V. parahaemolyticus* and *S. aureus* were reinoculated into 25 ml of mLB or LB broth at 30 and 37 °C and grown to OD_600_ of 0.8 respectively. The cultures were further incubated for 3 h with shaking (250 rpm) in the presence or absence of IPBC (50 µg/mL). After incubation, RNase inhibitor (RNAlater, Ambion, TX, United States) was added and chilled in dry ice bath containing 95% ethanol for 20 s to avert RNA degradation. Pellets were harvested (13,000 rpm; 5 min; 4 °C) and the total RNA using a Qiagen RNeasy mini-Kit (Valencia, CA, United States) was extracted. Note that the cell lysis was enhanced with the presence of acid-washed glass beads (Sigma-Aldrich, 150–212 μm, ~ 150 μL) added to the lysis buffer. qRT-PCR was performed using an SYBR™ Green qPCR Master Mix (Applied Biosystems, Foster City, United States) and an ABI StepOne Real-Time PCR System (Applied Biosystems). The cycle threshold values (Ct) for the genes were generated while 2^−ΔΔCT^ was employed to determine the relative gene expression level. Primers utilized for *V. parahaemolyticus* and *S. aureus* are updated in Supplementary Tables S1 and S2, respectively, while *16s rRNA* served as the endogenous control in both pathogens. The changes of each gene expression were determined using three independent cultures and six reactions per gene.

### Predictions of absorption, distribution, metabolism, and excretion (ADME) properties

The drug-likeness characteristics of iodopropynyl butylcarbamate (IPBC) were studied using the preADMET (https://preadmet.qsarhub.com/), molinspiration (https://www.molinspiration.com), and GUSAR (http://www.way2drug.com/gusar/) online platforms (all accessed on 14th April 2023). Regarding Lipinski's rule of five, "an orally active drug should have a molecular weight of ≤ 500 g/mol, a log P of ≤ 5, ≤ 5 hydrogen bond-donating atoms, ≤ 10 hydrogen-bond-accepting atoms, and an octanol–water partition coefficient of ≤ 140 Å2"^36^.

### Statistical analysis

Two independent cultures and three replicates were used for all experiments, and results are presented as means ± SDs. The Student's t-test was employed to analyze the significances of differences, and statistical significance was accepted for *P* values < 0.05.

## Results

### Screening of iodinated hydrocarbons against *V. parahaemolyticus* and *S. aureus* for their antibacterial and antibiofilm effects

The 22 iodinated hydrocarbons were evaluated for their abilities to inhibit planktonic cell growth and biofilm formation by *V. parahaemolyticus* and *S. aureus* at 100 µg/mL. Of the 22 candidate compounds, iodoform and iodopropynyl butylcarbamate (IPBC) exhibited significant activity against both pathogens **(**Supplementary Table S3**)**. In particular, iodoform inhibited the *V. parahaemolyticus* planktonic growth and biofilm formation by 81 and 60%, respectively, but those of *S. aureus* by only 17 and 7%, respectively. At the same dose, IPBC completely inhibited *V. parahaemolyticus* but inhibited *S. aureus* biofilm formation and cell growth by 73 and 92%, respectively. Regarding other compounds, 1, 2-diiodoethane inhibited *V. parahaemolyticus* and *S. aureus* biofilm formation by 20 and 24%, respectively, while 1-iodopropane and 2-iodopropane inhibited *S. aureus* biofilm formation by 20 and 21%, respectively (Supplementary Table S3).

**Figure 1:**
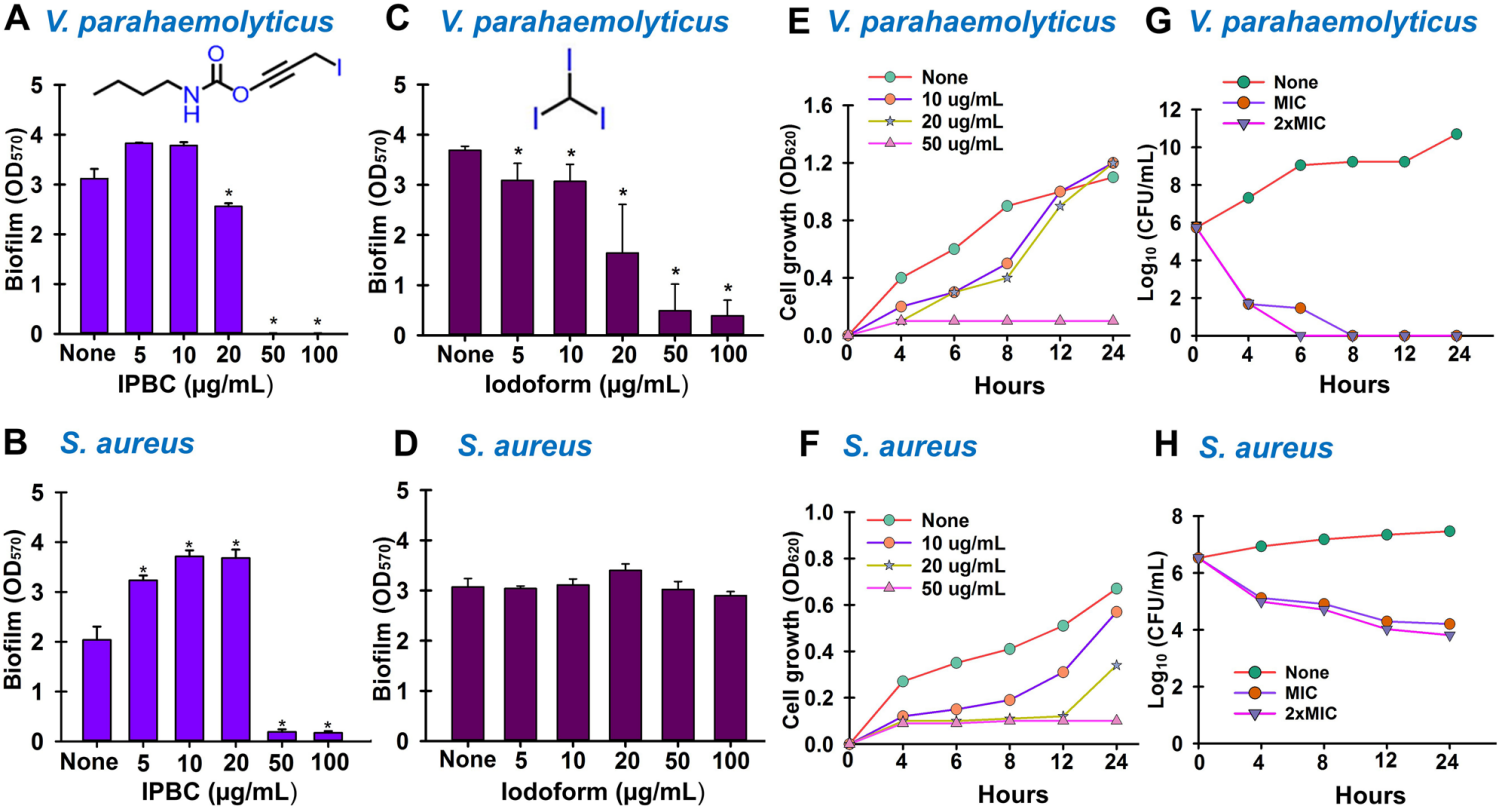
The effects of IPBC on *V. parahaemolyticus* and *S. aureus* biofilm formation (**A**, **B**), effects of iodoform on *V. parahaemolyticus* and *S. aureus* biofilm formation (**C**, **D**), effects of IPBC on planktonic cell growth (**E**, **F**) and the time-to-kill studies of IPBC against *V. parahaemolyticus* and *S. aureus* (**G**, **H**) *Significant difference at p < 0.05, and error bars represent standard deviations.

Due to the excellent inhibitory effects displayed by IPBC against both pathogens, its anti-biofilm, anti-cell growth, and anti-virulence effects were further examined. IPBC achieved 20% biofilm inhibition at 20 µg/mL and total inhibition at 50 µg/mL against *V. parahaemolyticus* and a similar effect against *S. aureus* at 50 µg/mL (Fig. 1A,B). This represented the minimum inhibitory concentration (MIC) for both pathogens, which suggested that the antibiofilm activities of IPBC were due to its cell growth inhibitory effects. Of note, IPBC increased biofilm formation at sub-MICs of 5–20 µg/mL against *S. aureus* (Fig. 1B). Furthermore, iodoform reduced *V. parahaemolyticus* biofilm formation by 20–60% and ˃ 85% at 5–20 µg/mL and 50–100 µg/mL, respectively (Fig. 1C). However, no antibiofilm activity was observed against *S. aureus* at the same doses of iodoform (Fig. 1D).

We also investigated the ability of IPBC to disperse established *V. parahaemolyticus* and *S. aureus* biofilms. While it had no significant eradicating effect on *S. aureus* preformed biofilms, IPBC dispersed 15% and 75% of *V. parahaemolyticus* preformed biofilms at 200 and 300 µg/mL, respectively. These concentrations were four to six times higher than that required to inhibit biofilm formation, which showed biofilm dispersal is much more difficult than biofilm inhibition (Supplementary Fig. S1).

### Effects of IPBC on planktonic cell growth

The growths of IPBC-treated cells were examined over 24 h to investigate the effect of IPBC on planktonic cells. The results showed that IPBC did not inhibit *V. parahaemolyticus* growth at 10 or 20 µg/mL but prevented its planktonic growth at 50 µg/mL. Similarly, at 50 µg/mL, IPBC completely inhibited *S. aureus* growth but had slight inhibitory effects, which did not impact biofilm inhibition potentials at 10–20 µg/mL (Fig. 1E,F). We also examined the killing efficacy of IPBC against both pathogens. As compared to the untreated control (6log_10_ CFU/mL), IPBC reduced *Vibrio* cell numbers by 4log_10_ at MIC and 2xMIC after 4 h. Total reduction (5log_10_) was achieved at MIC and 2xMIC after 6 and 8 h, respectively, indicating a bactericidal effect. Similarly, IPBC had a bactericidal effect on *S. aureus*. Specifically, IPBC at MIC and 2xMIC reduced *S. aureus* cells by 1log_10_ after 4 h and 2log_10_ after 24 h (Fig. 1G,H).

### Microscopic observations of biofilm formation by *V. parahaemolyticus* and *S. aureus*

**Figure 2:**
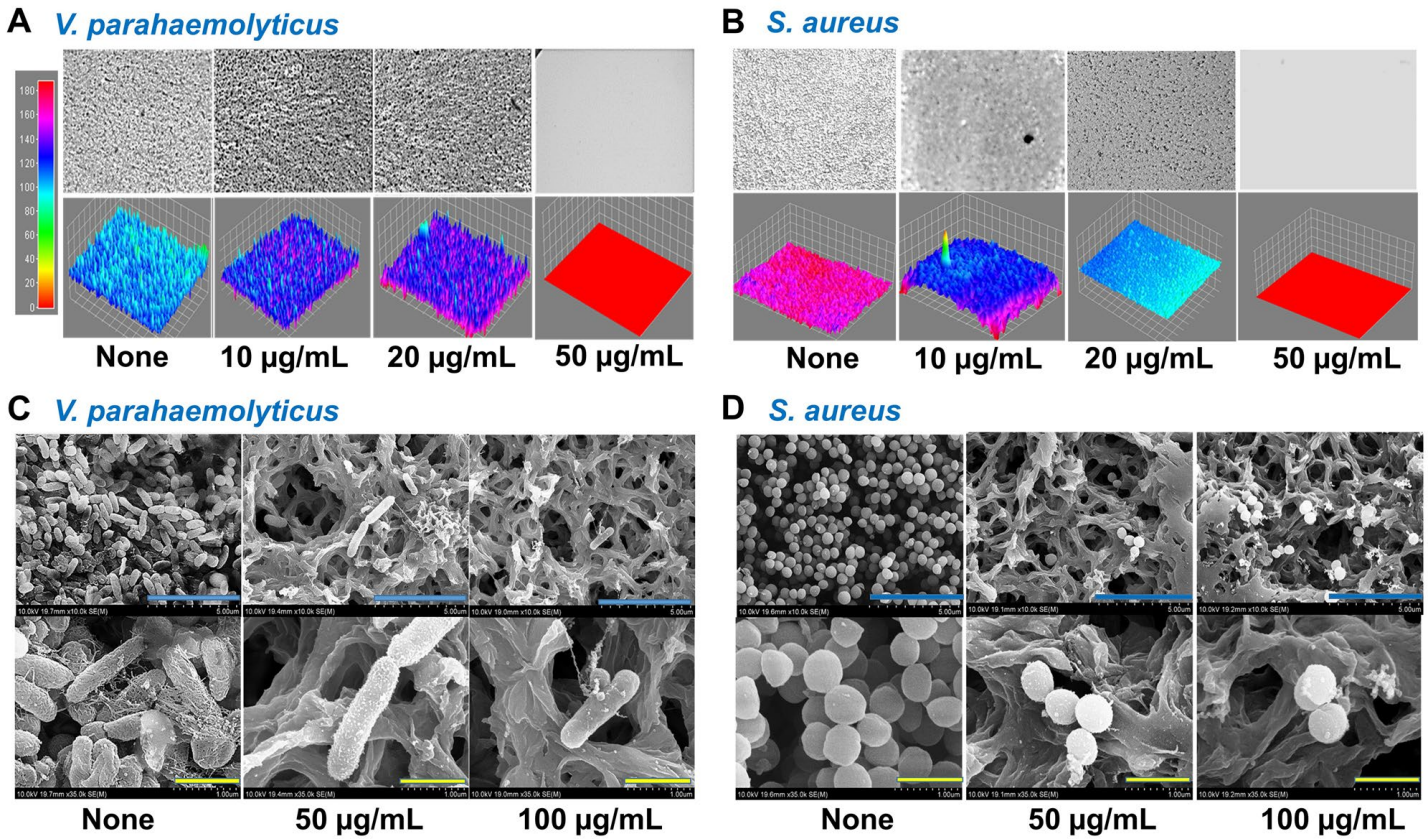
Microscopic studies of the antibiofilm effects of IPBC on *V. parahaemolyticus* and *S*. *aureus*. Live microscope color-coded 2D and 3D images of *V. parahaemolyticus* (**A**) and *S*. *aureus* (**B**) and SEM images of treated *V. parahaemolyticus* (**C**) *S*. *aureus* (**D**). Blue and yellow lines represent 5 and 1 µm, respectively.

3-D reconstructions of live microscopic images showed the presence of high biofilm biomasses in the untreated and 10–20 µg/mL treated groups and this was totally prevented at 50 µg/mL for both bacteria (Fig. 2A,B). SEM observations further confirmed these antibiofilm activities as fewer cell clusters were observed in 50–100 µg/mL treated samples than in untreated controls (Fig. 2C,D). Notably, at these doses, signs of membrane interference, such as shrinkage and bleb formation, were observed.

### IPBC inhibited *V. parahaemolyticus* motility

**Figure 3:**
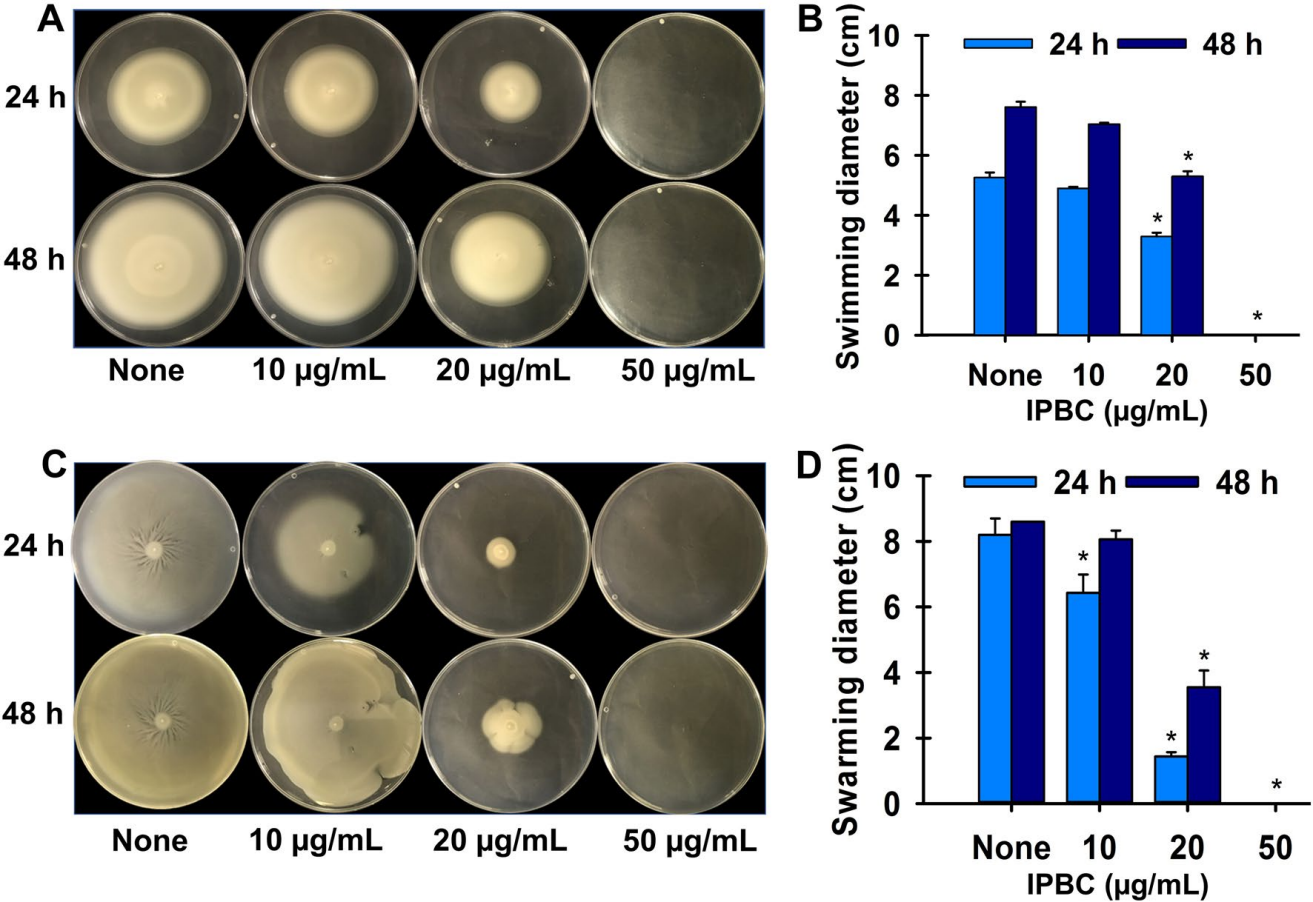
Effect of IPBC on the motilities of *V. parahaemolyticus*. (**A**) Effect on swimming motility, (**B**) swimming diameter measurements, (**C**) effect on swarming motility, and (**D**) swarming diameter measurements. *Significant difference at p < 0.05, while error bars represent standard deviation.

*Vibrio parahaemolyticus* has several advantages that enable it to form biofilms on seafood. In particular, its biofilm formation is assisted by a dual flagellar system involving polar and lateral flagella^4^. Therefore, we investigated the effects of IPBC on the swimming and swarming motilities of *V. parahaemolyticus* (Fig. 3). Interestingly, IPBC suppressed both swimming and swarming motilities of *V. parahaemolyticus* at a sub-MIC of 20 µg/mL and completely restricted movement when treated at 50 µg/mL for 48 h. In addition, IPBC had a greater inhibitory effect on the quorum sensing mediated swarming phenotype than on the swimming phenotype (Fig. 3A–D).

### Effects of IPBC on cell hydrophobicity and the hemolytic potentials of *V. parahaemolyticus* and* S. aureus*

**Figure 4:**
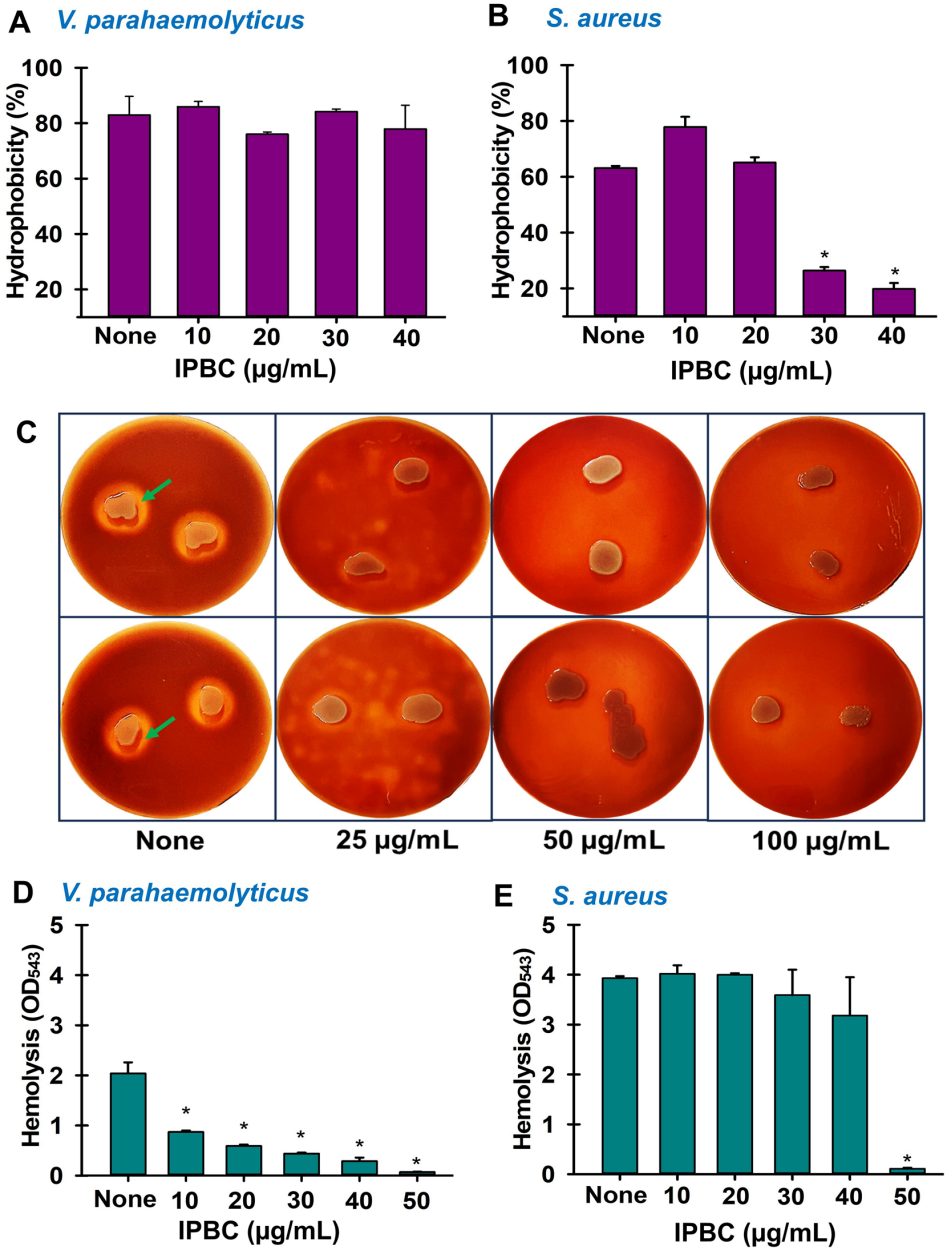
Effect of IPBC on virulence factors viz; cell surface hydrophobicity (**A**, **B**), *Vibrio* Kanagawa phenomenon on solid media (**C**) and the hemolysin production of *V. parahaemolyticus* and *S. aureus* in liquid media (**D**, **E**). *Significant difference at p < 0.05, and error bars represent standard deviations. Green arrows represent the hemolysis typical of *Vibrio* thermostable direct hemolysin (TDH).

Microbial outer membranes harbor a number of compounds that enable cell attachment to surfaces, and thus, disrupting these activities provides a possible means of preventing surface colonization and biofilm formation. Hence, we investigated the effect of IPBC on cell surface hydrophobicity (CSH) which influences the ability of bacteria to colonize different surfaces. IPBC did not inhibit *V. parahaemolyticus* CSH at 10–40 µg/mL but did reduce S. *aureus* CSH by 58 and 66% at sub-MICs of 30 and 40 µg/mL, respectively (Fig. 4A,B).

*Vibrio parahaemolyticus* produces thermostable direct hemolysin and other related hemolysins which are central to its pathogenicity. Similarly, hemolysin production was reported to be crucial for the virulence of *S. aureus* and the main cause of the β-hemolytic phenotype in *S. aureus*. Therefore, we examined the ability of IPBC to inhibit hemolysin production in both bacteria. Interestingly, IPBC at ≥ 25 µg/mL visibly prevented the formation of clear halo zone indicative of *Vibrio* Kanagawa phenomenon (Fig. 4C). A similar trend was observed in the liquid media where IPBC dose-dependently reduced the hemolytic capacity of *V. parahaemolyticus* at sub-MICs 10–40 µg/mL by 40–80% (Fig. 4D). However, it had no significant effect on *S. aureus* hemolysin production at < 40 µg/mL except at higher concentrations which killed the bacteria (Fig. 4E). Similarly, staphyloxanthin production, a carotenoid pigment believed to be responsible for *S. aureus* protection against innate immunity was not perturbed by the activity of IPBC at sub-MICs (Supplementary Fig. S2).

### Antibiofilm activities of IBPC against other pathogens

**Figure 5:**
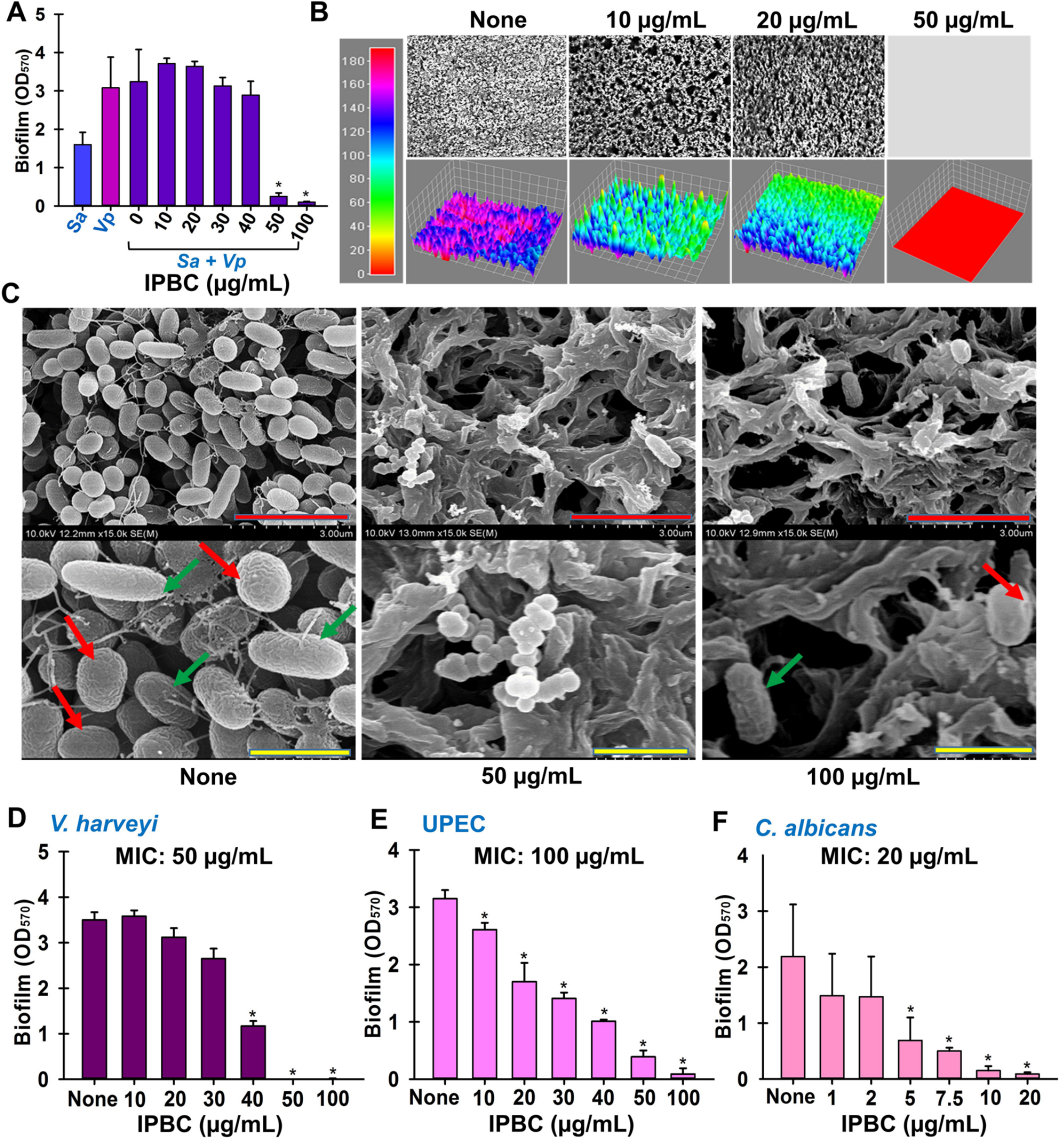
The effects of IPBC on mixed species biofilm involving *V. parahaemolyticus* (*Vp*) and *S. aureus*—*Sa* (**A**), live imaging microscopic examination remodeled as color coded 3D images of the mixed species biofilm interaction (**B**), SEM images of mixed biofilms (**C**) and the antibiofilm activities against *V. harveyi* (**D**), UPEC (**E**) and *Candida albicans* (**F**). *Significant difference at p < 0.05 and error bars represent the standard deviation. Red and yellow scale bars denote 3 and 1 µm while the red and green arrows represent *S. aureus* and *V. parahaemolyticus* respectively.

Bacteria usually exist in mixed communities, and this is especially true in food production facilities where the interaction is pronounced due to nutrient availability. Accordingly, we optimized suitable conditions for dual species biofilm formation (Supplementary Fig. S3) and examined the effects of IPBC on mixed *V. parahaemolyticus*/*S. aureus* biofilm formation. As was observed for single biofilm inhibition, IPBC totally inhibited dual biofilm formation at the same dose of 50 µg/mL (Fig. 5A). Furthermore, at 50 and 100 µg/mL IPBC totally disrupted biofilm biomass of mixed *S. aureus* and *V. parahaemolyticus* biofilms. Similar to the single species interaction (Fig. 2C,D), the live microscopy and SEM studies showed the membrane-disrupting capacities of the IPBC against the pathogens in addition to preventing micro colony formation at 100 µg/mL (Fig. 5B,C).

In addition, the broad-spectrum antimicrobial and antibiofilm effects of IPBC were examined against *V*. *harveyi*, uropathogenic *E. coli* (UPEC), and *C. albicans*. Interestingly, IPBC inhibited *V. harveyi*, which is responsible for vibriosis and enormous economic losses in the aquaculture industry by 60% at sub-MIC of 40 µg/mL and MIC (50 µg/mL) **(**Fig. 5D). Also, UPEC has been reported to colonize the colon and cause urinary tract infections, whereas *C. albicans* is a known cause of candidiasis, oral thrush, and genital and urinary yeast infections. In this study, IPBC had MICs of 100 and 20 µg/mL against UPEC and *C. albicans,* respectively, dose-dependently inhibited their biofilm formations at sub-MICs and completely inhibited their biofilm formations at 100 and 20 µg/mL, respectively (Fig. 5E,F). Overall, *C. albicans* biofilm was more susceptible to IPBC than *V. harveyi* and UPEC.

### IBPC inhibited *V. parahaemolyticus* and *S. aureus* in shrimp model

**Figure 6:**
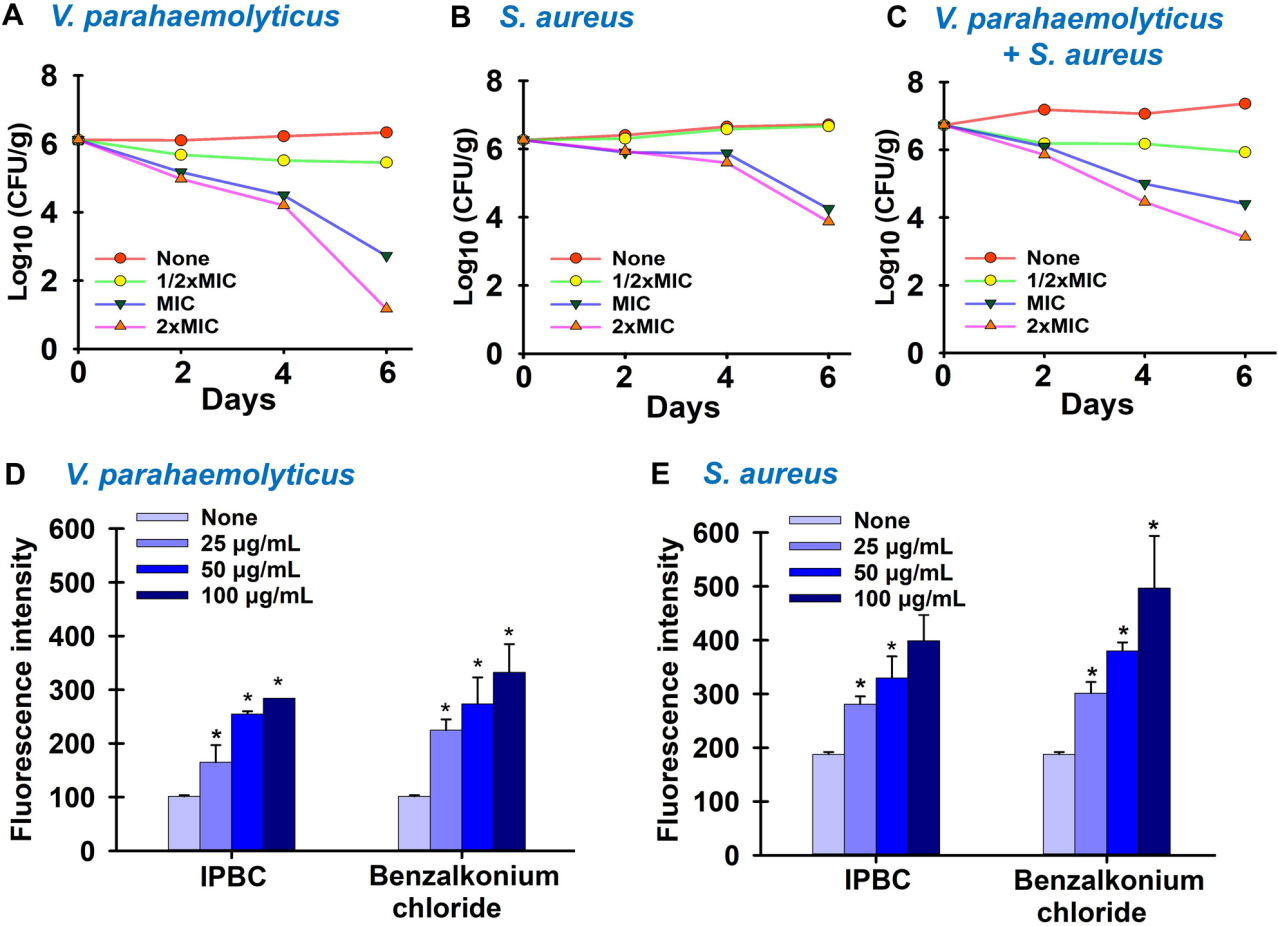
Effects of IPBC on the proliferation of *V. parahaemolyticus* (**A**), *S. aureus* (**B**) and both pathogens (**C**) in a shrimp model and its effects on cell membrane permeabilities of *V. parahaemolyticus* (**D**) and *S. aureus* (**E**) as determined by the NPN uptake assay. *Significant difference at p < 0.05 and error bars represent the standard deviation.

*Vibrio parahaemolyticus* and *S. aureus* were previously isolated together from seafood products due to intrinsic food properties or cross-contamination during preparation^37^. Therefore, IPBC was investigated as a potential alternative to control these pathogens in a shrimp model. The result showed that IPBC inhibited the proliferation of *S. aureus* by ~ 2log_10_ at MIC and 2 × MIC after 6 days while the *Vibrio* load was reduced by a similar value after 4 days. It however became bactericidal with ˃ 4log_10_ reduction after 6 days (Fig. 6A,B). Notably, the presence of both pathogens on the inoculated shrimp was diminished by ~ 2.5 and 3log_10_ at MIC and 2 × MIC respectively (Fig. 6C).

### Effect of IPBC on the cell wall integrities of *V. parahaemolyticus and S. aureus*

Sequel to the membrane disruption by IPBC as revealed by the SEM studies (Fig. 5C), the effects of IPBC on the bacterial membrane were biochemically analyzed. *N*-Phenyl-napthylamine (NPN) is a hydrophobic dye which emits high fluorescence in hydrophobic environments and can penetrate hydrophobic environments inside cell membranes^27^. In this study, IPBC at ½ × MIC, MIC, and 2 × MIC significantly increased the fluorescence intensity of NPN in both bacteria, indicating increased NPN penetration due to compromised cell walls (Fig. 6D,E). Notably, the effect of benzalkonium chloride (a known cell wall disruptor and positive control) was more pronounced than that of IPBC.

### IPBC repressed genes related to biofilm formation and virulence

**Figure 7:**
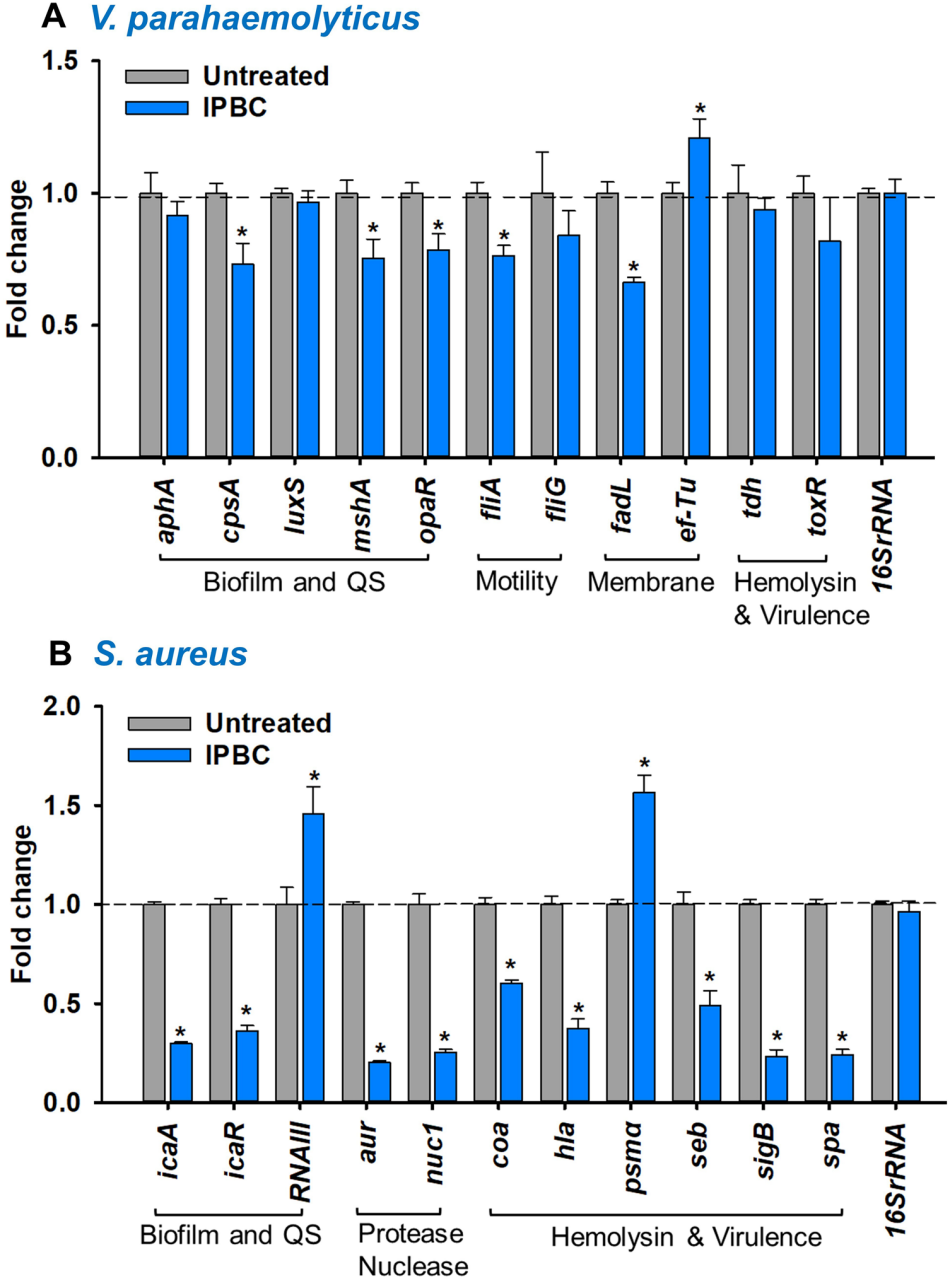
The effects of IPBC treatment (50 µg/mL) on the expressions of genes related to biofilm formation and virulence activities in *V. parahaemolyticus* (**A**) and *S. aureus* (**B**). *Significant difference at p < 0.05 and error bars represent the standard deviation.

To further elucidate the basis underlying the various activities of IPBC, its effect on expressions of eleven biofilm- and virulence-related genes in each pathogen were examined. In *V. parahaemolyticus*, IPBC downregulated *cpsA*, *mshA, opaR, fliA* and *fadL* genes by 1.3–1.5-fold but upregulated *ef-Tu* while others including *aphA*, *luxS*, *fliG*, *tdh* and *toxR* related to quorum sensing (QS) and virulence were not significantly affected (Fig. 7A). Also, IPBC significantly repressed biofilm-, virulence- and toxin-related genes including *icaA*, *icaR*, *aur*, *nuc1*, *hla*, *seb*, *sigB* and *spa* in *S. aureus* by 3.4-, 2.8-, 5.0-, 3.9-, 2.7-, 2.0-, 4.3- and 4.1-fold, respectively. However, it upregulated *RNAIII* and *psmα* related to QS and virulence by 1.5 and 1.6-fold, respectively (Fig. 7B).

### ADME profiling of IPBC

IPBC was subjected to ADME analysis to predict its toxicological, physicochemical, and bioavailability properties and found not to contravene Lipinski's rule of five or other standards, including Veber's and Muegge's parameters, which are used by GSK and Bayer, respectively (Supplementary Table S4). ADME analysis showed IPBC has good lipophilicity, water solubility, and high gastrointestinal, skin, and brain barrier permeability without acute fish toxicity. Furthermore, the toxicity of IPBC was found to fall within the applicability domain of rat models and it is a non-inhibitor of CYP1A2 and CYP3A4 which is responsible for drug metabolism, but IPBC showed a tendency for carcinogenicity in rats.

## Discussion

Biofilm formation remains an important source of surface or device contamination in food and clinical environments leading to infection, increased spoilage, and persistence against an array of treatments. Biofilm production by *V. parahaemolyticus* and *S. aureus* on food and clinical devices, and their biofilm-conferred antibiotic resistances have been well reported^12,38^. In this study, IPBC displayed the most potent antimicrobial and biofilm inhibitory activities against *V. parahaemolyticus* and *S. aureus* at 50 µg/mL among the 22 iodinated hydrocarbons investigated (Supplementary Table S3, Fig. 1A).

Of the 22 iodine-containing materials examined, the antibiofilm activities of IPBC and iodoform were attributed mainly to growth inhibition (Fig. 1E,F). Although no study has investigated the antibiofilm effects of IPBC, its antibacterial activity, as observed in the present study, is consistent with previous reports on its inhibition of *S. epidermidis*, *S. capitis*, *M. luteus*, *Bacillus*, *Acinetobacter*, *P. aeruginosa*, A*spergillus niger*, *Penicillium citrintim* and *C. albicans*^39,40^. In addition, iodoform, a well-known antiseptic, dose-dependently inhibited *V. parahaemolyticus* biofilm formation (Fig. 1C,D), which is in line with its reported antibacterial effects against *E. coli*^41^ and inhibition of mixed *Enterococcus faecalis* biofilm formation^42^.

In the current study, IPBC significantly eradicated preformed *V. parahaemolyticus* biofilms at 300 µg/mL, which was 6 times higher than its MIC (50 µg/mL), but not preformed *S. aureus* biofilms (Supplementary Fig. S1). This result confirms the difficulty of mature biofilm treatments and also indicates IPBC can penetrate, and control established *V. parahaemolyticus* biofilms more effectively than established *S. aureus* biofilms. Furthermore, the higher bactericidal effects of IPBC on *V. parahaemolyticus* than *S. aureus* (Fig. 1G,H) at 2 × MIC (5 log and 2 log CFU/mL reduction, respectively) corroborates its pronounced biofilm eradicating effect on *V. parahaemolyticus* than *S. aureus* (Supplementary Fig. S1). SEM observations further validated the efficacy of IPBC as evidenced by the absence of microcolonies, which indicated the suppression of extracellular polymeric substance (EPS) production essential for protection or surface attachment (Fig. 2B). SEM also revealed that IPBC may have disrupted the cell membranes of *S. aureus* and *V. parahaemolyticus* (Fig. 5C), and the enhanced NPN membrane uptake which is similar to the effect of benzalkonium chloride obtained in this study supported this observation (Fig. 6D,E). Our findings corroborates a previous study where IPBC exhibited fungicidal activity by interacting with membrane fatty acids to alter membrane permeability^43^.

Furthermore, the quality and economic value of uncooked seafoods are threatened by microbial contamination including *V. parahaemolyticus* and *S. aureus*^10^. Notably, IPBC inhibited the proliferation of the pathogens separately and together on shrimp (Fig. 6A–C). This is consistent with previous study that *Lachnum* YM30 melanin inhibited both pathogens^44^. Similarly, extracts from pomegranate and Chinese gall inhibited *V. parahaemolyticus* and *Listeria monocytogenes* occurrence on raw tuna and cooked shrimp^45^. Therefore, our observation suggests the capacity of IPBC to prevent *Vibrio* or Staphylococcal mediated biodeterioration and thus could serve as a preservative. Also, IPBC drastically reduced the mixed *V. parahaemolyticus*/*S. aureus* biofilm formation and inhibited the planktonic and biofilm cells of medically important pathogens, such as *V*. *harveyi*, UPEC, and *C. albicans*, with low MICs (20–100 µg/mL). This is in line with the previously reported inhibitory activities of wipes containing IPBC against *S. aureus, Acinetobacter baumannii,* and *Clostridium difficile* spores^46^. Similarly, IPBC loaded on halloysite showed excellent antifungal activities against *Aspergillus niger, Penicillium citrintim, Trichoderma viride,* and *Botryodiplodia theobromae*^47^. Thus, our findings confirm that IPBC is a broad-spectrum antimicrobial agent against fungi, Gram-positive and Gram-negative bacteria with potential use as a disinfectant, surfactant, or preservatives for controlling biofilms in food and clinical settings.

Regarding antivirulence potentials, the presence of flagellum, pili, and fimbriae influences the degree of biofilm formation and biofilm morphology^15^. Our motility assays showed that IPBC reduced flagella production, and thus, limited the ability of *Vibrio* to colonize liquid and solid surfaces (Fig. 3). Additionally, QS controls the swarming potential of *V. parahaemolyticus*^6^, and the suppression of swarming by IPBC suggests an ability to interfere with QS systems at the sub-MIC level. Furthermore, sub-MIC IPBC reduced the cell surface hydrophobicity of *S. aureus,* and thus, its ability to attach to hydrophobic surfaces (Fig. 4B). Considering the correlation between CSH and biofilm formation^48^ and its inhibition by sub-MICs of IPBC, we expected that IPBC would inhibit biofilm formation in *S. aureus* at similar doses but was found to have no effect (Fig. 1B), and this implies that CSH may not primarily influence biofilm formation in *S. aureus*. In addition, IPBC could not inhibit *Vibrio* CSH at concentrations ≤ 40 µg/mL, which suggests it may target *V. parahaemolyticus* attachment properties other than CSH as observed in motility inhibition (Fig. 3).

The thermostable toxin associated with blood lysis, fluid accumulation, and mammalian cytotoxicity was inhibited by IPBC at sub-MICs in both solid (Kanagawa phenomenon) and liquid media (Fig. 4C,D), indicating that IPBC might deactivate the invasiveness associated with hemolysin secretion in *V. parahaemolyticus*. This result corroborates a previous report in which povidone iodine inhibited virulence factors, including endotoxins, lipase, and elastase in *E. coli* and *P. aeruginosa*^49^. Unlike its activity in *Vibrio*, IPBC lacked antihemolytic activity against *S. aureus* at sub-MICs, indicating an inability to curb associated membrane damage and cell death. Overall, in addition to its antibiofilm and bactericidal activities, IPBC differentially inhibited the motility, hemolysis, and the cell surface hydrophobicity of *V. parahaemolyticus* and *S. aureus*.

IPBC is an iodine-containing carbamate approved by the Food and Drug Administration (FDA) in food packaging and storage as an adhesive component^50^. It is also used as a preservative in cosmetic products^18^. We speculate that an interaction between the iodine atom and the alkyne bond present on the carbamate scaffold potentiates the activity of IPBC against *S. aureus* and *V. parahaemolyticus.* This suggestion is partially supported by the membrane disruption typical of iodine observed in this study and the reported antimicrobial effects of cepacin and caryoynecin, which both contain triple alkyne bonds, against *S. aureus*, *E. coli,* and *Klebsiella pneumonia*^51^.

Furthermore, we investigated the effects of IPBC on the expression of biofilm- or virulence-related genes to gain possible mechanistic insight. In *V. parahaemolyticus*, the expression of *cpsA* involved in capsular polysaccharide production for biofilm formation^52^, the mannose-sensitive hemagglutinin (*mshA*), *fliA* and *opaR* encoding type IV pili, flagella sigma factor and QS, respectively^7,53^ were repressed (Fig. 7A), which partially explains the inhibitory effect of IPBC on biofilm formation and virulence factors. However, despite the significant phenotypic inhibition of swimming, swarming and hemolysin production even at lower doses (Figs. 3, 4C,D), IPBC displayed a slight or insignificant downregulation of responsible genes including *fliG* and *tdh*. Additionally, the downregulation of *fadL* gene acting as an outer membrane fatty acid transporter for active import of exogenous fatty acids and cell membrane maintenance^54^ corroborates the *Vibrio* membrane disruption displayed by IPBC (Fig. 5C). Despite the forgoing, it is worthy to note that the magnitude of gene downregulation reported may not be sufficient to establish a clear molecular mechanism for *Vibrio*. Hence, further molecular study is required to gain detailed insight.

In *S. aureus*, biofilm formation and survival are complicated by series of environmental factors, proteases, global regulators, surface proteins, and QS^55^. IPBC significantly repressed the transcription levels of different genes including *icaA*, *aur*, *hla*, and *spa* (Fig. 7B). Briefly, *icaA* is a key component of *S. aureus* adhesion locus responsible for the production of polysaccharide intercellular adhesin and needed for biofilm formation^56^. The surface binding protein A (*spa*) gene accounts for *S. aureus* evasion of the immune system phagocytosis and plays an important role in *S. aureus* biofilm formation^57,58^. Also, the *hla* gene is essential for *S. aureus* deadly α-hemolysis and contributes to biofilm formation^59^. Their inhibitions suggest a molecular explanation for the observed antibiofilm and antivirulence activities of IPBC. However, the major effector molecule of *S. aureus* QS system (*RNAIII*) and phenol soluble modulin (*psmα*) were upregulated by IPBC treatment. The presence of PSMα was reported to enhance biofilm dispersal and induces strong *S. aureus* aggregation at low concentration^60^. Hence, the upregulation of PSMα upregulation partially supports biofilm inhibition. In all, the repression of various genes in both pathogens (Fig. 7) further attests to the possible mechanisms supporting the activities of IPBC.

IPBC displayed reasonable drug-likeness properties (Supplementary Table S4), which support the non-genotoxic, good skin penetration, and non-carcinogenic findings reported at lower doses in rats^61^. However, other toxicity concerns may be alleviated using nanotechnologies. Thus, it appears IPBC is suitable for further applications in clinical and food processing environments. For instance, it could be utilized in combination with several FDA-approved drugs, such as trifluoperazine to potentiate activities as previously reported by^50^.

## Conclusion

In this study, IPBC demonstrated effective inhibitory activities against planktonic *V. parahaemolyticus* and *S. aureus* cells and biofilm formation by these cells. Virulent traits, including motility, hemolysin production, and cell surface hydrophobicity associated with surface attachment and biofilm formation, were inhibited by IPBC. Furthermore, IPBC suppressed mixed *S. aureus*/*V. parahaemolyticus* biofilms, exhibited preservative potentials in a food model and inhibited biofilm formation by other food and clinically relevant pathogens, including UPEC, *C. albicans*, and *V. harveyi*. The current study further revealed that IPBC damaged cell membranes and downregulated responsible genes to exhibit the antibiofilm and antivirulence properties. Overall, it seems that when utilized as an antibacterial or antibiofilm agent, IPBC offers a viable alternative for the control *S. aureus* and *V. parahaemolyticus* in food or clinical facilities. We suggest detailed in vivo studies be conducted to confirm the suitability of IPBC for these end uses.

### Supplementary Information

Supplementary Information.

## Data Availability

All data generated or analyzed in this present study are included in this published article or its supplementary information files.
