# Supplementary material for: Antibacterial and antibiofilm activities of iodinated hydrocarbons against Vibrio parahaemolyticus and Staphylococcus aureus
Source: Sci Rep. 2024 Apr 22;14:9160. doi: 10.1038/s41598-024-55479-7 (PMC11033260; doi:10.1038/s41598-024-55479-7)
Supplement: Supplementary file 1 — Supplementary Information. [file 41598_2024_55479_MOESM1_ESM.docx]

**Supplementary information**

**Antibacterial and Antibiofilm Activities of Iodinated** **Hydrocarbons against *Staphylococcus aureus* and *Vibrio parahaemolyticus***

Oluwatosin Oluwaseun Faleye^§^, Olajide Sunday Faleye^§^, Jin-Hyung Lee, and Jintae Lee*

School of Chemical Engineering, Yeungnam University, 280 Daehak-Ro, Gyeongsan, 38541,

Republic of Korea

^§^These authors contributed equally to this work.

*Corresponding Author

E-mail: jtlee@ynu.ac.kr. Tel.: +82-53-810-2533. Fax: +82-53-810-4631.

**Supplementary Table S1.** Primers used in qRT-PCR for *Vibrio parahaemolyticus.*

| **Genes** | **Functions** | **Primers Sequence, Forward/Reverse** |
| --- | --- | --- |
| *aphA* | Acid phosphatase AphA; biofilm, motility | 5’-ACACCCAACCGTTCGTGATG-3’ |
|  |  | 5’-GTTGAAGGCGTTGCGTAGTAAG-3’ |
| *cpsA* | Capsular polysaccharide | 5’-GCGCACAACGAAGAATATCG-3’ |
|  |  | 5’-CCATCTTATCGAGCGTGTCG-3’ |
| *luxS* | Autoinducer binding domain-containing protein; QS; biofilm | 5’-GAT GGG ATG TCG CAC TGG TTT-3’ |
|  |  | 5’- ACT TGC TGT TCA GAA GGC GTA-3’ |
| *mshA* | Type IV pilin MshA | 5’-GGTTTCGTTTAGGTCACG -3’ |
|  |  | 5’-CGTCGAAATGTCGGCGG-3’ |
| *opaR* | Transcriptional regulator OpaR | 5’-TGTCTACCAACCGCACTAACC-3’ |
|  |  | 5’-GCTCTTTCAACTCGGCTTCAC-3’ |
| *fliA* | Flagellar biosynthesis sigma factor | 5’-TAAGCGTATTGCTCACCACCT-3’ |
|  |  | 5’-GCTCGCACCTTTAGAACCAT-3’ |
| *fliG* | Flagellar motor switch protein G | 5’-TTCTGCTGGGTACTGGTTCG-3’ |
|  |  | 5’-ATTTGCGGGTGTTCGTTG-3’ |
| *fadL* | Long-chain fatty acid outer membrane channel/bacteriophage T2 receptor | 5’-ACGATAAAGGTCAGGAAATCAC-3’ |
|  |  | 5’-GTATTGGATGCTGTAATGTACGG-3’ |
| *vmeB* | Multidrug efflux RND transporter permease subunit VmeB | 5’-CTGCGACCATTACACTGACTT-3’ |
|  |  | 5’-GTGTGTAAAGTCTGGATCGTC-3’ |
| *tdh* | Thermostable direct hemolysin (TDH) | 5’-GTAAAGGTCTCTGACTTTTGGAC-3’ |
|  |  | 5’-TGGAATAGAACCTTCATCTTCACC-3’ |
| *toxR* | Controls virulence, fitness, effector and hemolysin production | 5’-TTGTTTGGCGTGAGCAAGG-3’ |
|  |  | 5’-TAGCAGAGGCGTCATTGTTATC-3’ |
| *16S rRNA* | Housekeeping gene | 5’-TATCCTTGTTTGCCAGCGAG-3’ |
|  |  | 5’-CTACGACGCACTTTTTGGGA-3’ |

**Supplementary Table 2.** Primers used in qRT-PCR for *Staphylococcus aureus*.

| Gene | Name | Primer |
| --- | --- | --- |
| *icaA* | Intercellular adhesion A | 5’-TGA ACC GCT TGC CAT GTG-3’ |
|  |  | 5’-CAC GCG TTG CTT CCA AAG A-3’ |
| *icaR* | Intercellular locus regulator | 5'-TCG AAC TAT TCA ATT GAT GCT TTA-3' |
|  |  | 5'-CAG AAA ATT CCT CAG GCG TA-3' |
| *RNAⅢ* | Transcriptional regulator | 5'-ATC GAC ACA GTG AAC AAA TTC AC-3' |
|  |  | 5'-CTC TAC TAG CAA ATG TTA CTC AC-3' |
| *aur* | Zinc metalloproteinase aureolysin | 5'-ACC GTG TGT TAA TTC GTG TGC TA-3' |
|  |  | 5'-ATG GTC GCA CAT TCA CAA GTT T-3' |
| *coa* | Coagulase | 5'-CAC GGA AAT GGC CAA GTA TC-3' |
|  |  | 5'-TCG GAC GAG CTC CAT ATG AT-3' |
| *hla* | α-Hemolysin | 5'-CGG CAC ATT TGC ACC AAT AAG GC-3' |
|  |  | 5'-GGT TTA GCC TGG CCT TCA GC-3' |
| *nuc1* | Nuclease | 5'-CAC CTG AAA CAA AGC ATC CTA A-3' |
|  |  | 5'-TAT ACG CTA AGC CAC GTC CAT-3' |
| *psmα* | Phenol soluble modulins α | 5'-ACC CAT GTG AAA GAC CTC CTT TGT-3' |
|  |  | 5'-ATG GGT ATC ATC GCT GGC ATC-3' |
| *seb* | Enterotoxin B | 5'-TGT TCG GGT ATT TGA AGA TGG -3'  5'-CGT TTC ATA AGG CGA GTT GTT-3' |
| *sigB* | RNA Polymerase sigma factor | 5'-AAG TGA TTC GTA AGG ACG TCT-3' |
|  |  | 5'-TCG ATA ACT ATA ACC AAA GCC T-3' |
| *spa* | Protein A | 5'-ACC AGA AAC TGG TGA AGA AAA TCC-3' |
|  |  | 5'-TAA CGC TGC ACC TAA GGC TAA TG-3' |
| *16S*  *rRNA* | A component of ribosomes | 5'-TGT TTG ACG ATG TTT GAG CA-3' |
|  |  | 5'-CCT TCC TCC AGT TCA GAT GC -3' |

**Supplementary Table 3.** Percentage (%) biofilm and cell growth inhibition of 22 iodinated hydrocarbons against *V. parahaemolyticus* and *S. aureus* at 100 µg/mL. *The % biofilm and cell growth inhibition is defined as the percentage reduction of biofilm formation and cell growth after treatment compared to the control.

| **Compounds** |  | ***V. parahaemolyticus*** | | ***S. aureus*** | |
| --- | --- | --- | --- | --- | --- |
|  | **Structures** | Biofilm  inhibition  (%) | Growth  inhibition  (%) | Biofilm inhibition  (%) | Growth inhibition  (%) |
| 1-Chloro-4-iodobutane | 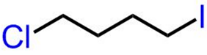 | 0 | 0 | 8 | 10 |
| 1-Chloro-6-iodohexane | 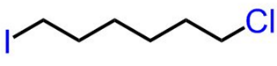 | 0 | 0 | 0 | 9 |
| Chloroiodomethane | 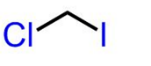 | 0 | 0 | 0 | 6 |
| 1-Chloro-3-iodopropane | 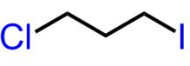 | 2 | 0 | 0 | 3 |
| 1,4-Diiodobutane | 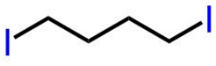 | 0 | 5 | 0 | 0 |
| 1,10-Diiododecane | 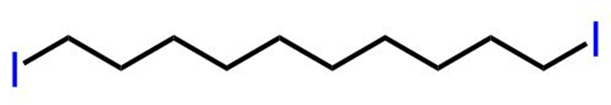 | 0 | 23 | 0 | 0 |
| 1,2-Diiodoethane | 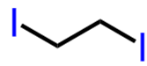 | 20 | 13 | 24 | 0 |
| 1,6-Diiodohexane | 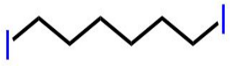 | 0 | 0 | 12 | 0 |
| Diiodomethane | 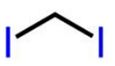 | 0 | 0 | 0 | 0 |
| 1,5-Diiodopentane | 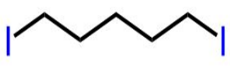 | 0 | 1 | 10 | 0 |
| 1,3-Diiodopropane | 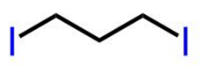 | 0 | 10 | 0 | 4 |
| 1-Iodododecane | 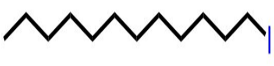 | 0 | 0 | 0 | 16 |
| Iodoethane | 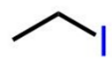 | 0 | 0 | 0 | 7 |
| Iodoform | 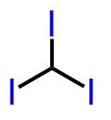 | 81 | 60 | 17 | 7 |
| 1-Iodoheptane | 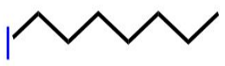 | 0 | 0 | 0 | 2 |
| 1-Iodohexane | 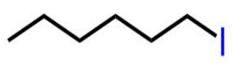 | 0 | 0 | 0 | 0 |
| 1-Iodononane | 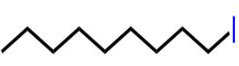 | 7 | 0 | 2 | 0 |
| 1-Iodooctane | 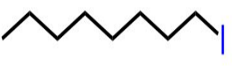 | 0 | 0 | 0 | 0 |
| 1-Iodopropane | 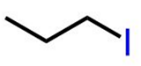 | 0 | 0 | 20 | 3 |
| 2-Iodopropane | 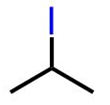 | 0 | 0 | 21 | 2 |
| 3-Iodopropanol | 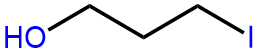 | 0 | 4 | 0 | 8 |
| Iodopropynyl butylcarbamate  (IPBC) |  | 100 | 100 | 73 | 92 |

**Supplementary Table 4.** Absorption, distribution, metabolism and excretion (ADME) properties of IPBC

| **Property** | **Iodopropynyl butylcarbamate** |
| --- | --- |
| Lipinski (Pfizer) rule of five | Suitable |
| Lipinski rule of five violations | 0 |
| Veber (GSK) rule | Suitable |
| Muegge (Bayer) rule | Suitable |
| Plasma protein binding | 97.959085 |
| Blood brain barrier permeability | 1.8801 (Yes) |
| Lipophilicity | 2.16 |
| Solubility (Log *S*) | Soluble (-2.52) |
| P-gp substrate | No |
| CYP1A2 inhibitor | No |
| CYP3A4 inhibitor | No |
| Skin permeability | -2.32484 |
| Gastrointestinal intestinal absorption | 96.7% (High) |
| Caco2 | 24.4269 |
| Mouse carcinogenicity | Positive |
| Rat carcinogenicity | Positive |
| Acute algae toxicity | 0.0326326 |
| Acute fish toxicity (medaka) | 0.0137451 |
| Acute fish toxicity (minnow) | 0.018077 |
| *In vitro* hERG inhibition | Low risk |
| miLogP | 2.84 |
| Mol volume | 182.91 |
| TPSA | 38.33 |
| GPCR ligand | -0.29 |
| Ion channel modulator | 0.04 |
| Kinase inhibitor | -1.02 |
| Nuclear receptor ligand | -0.69 |
| Protease inhibitor | -0.22 |
| Enzyme inhibitor | 0.30 |
| Rat IP LD50 classification | Class 4 in AD |
| Rat IV LD50 classification | Class 4 in AD |
| Rat oral LD50 classification  Rat SC LD50 classification | Class 4 in AD  Class 4 in AD |


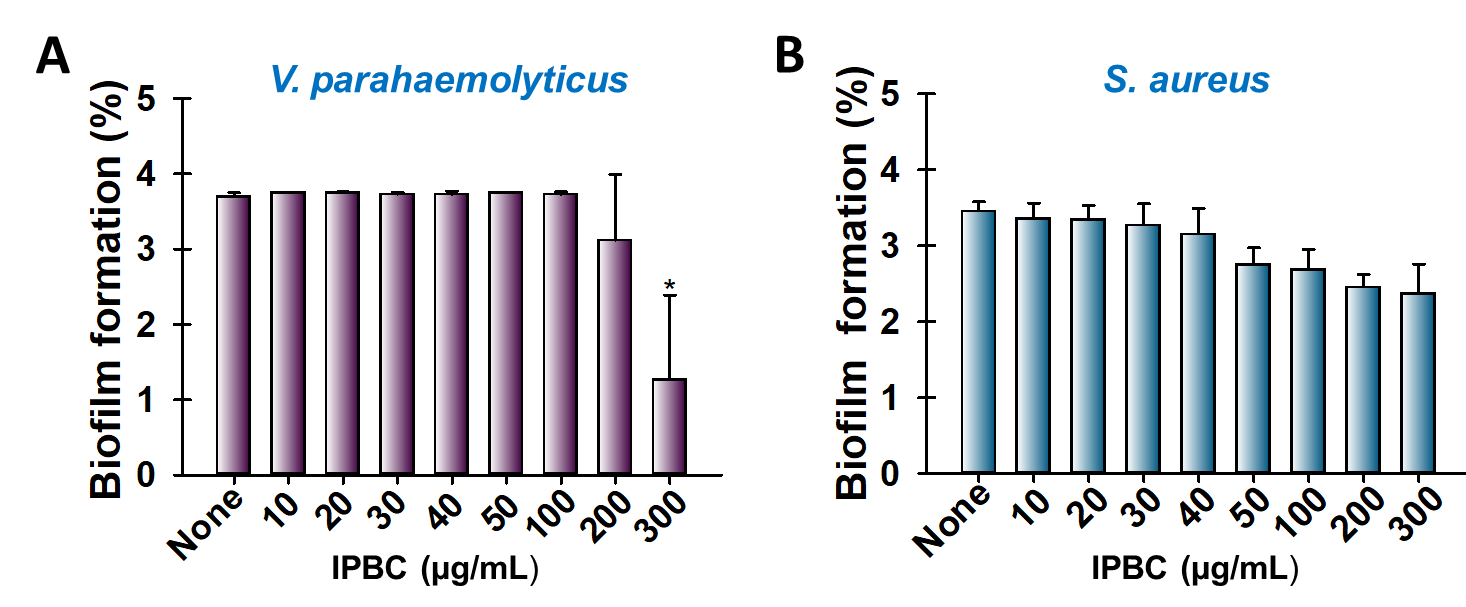


**Supplementary Figure 1.** The biofilm disrupting effects of IPBC on preformed biofilm of *V. parahaemolyticus* (A) and *S*. *aureus* (B) Biofilms were established for 24 h and IPBC was added and incubated for another 24 h.


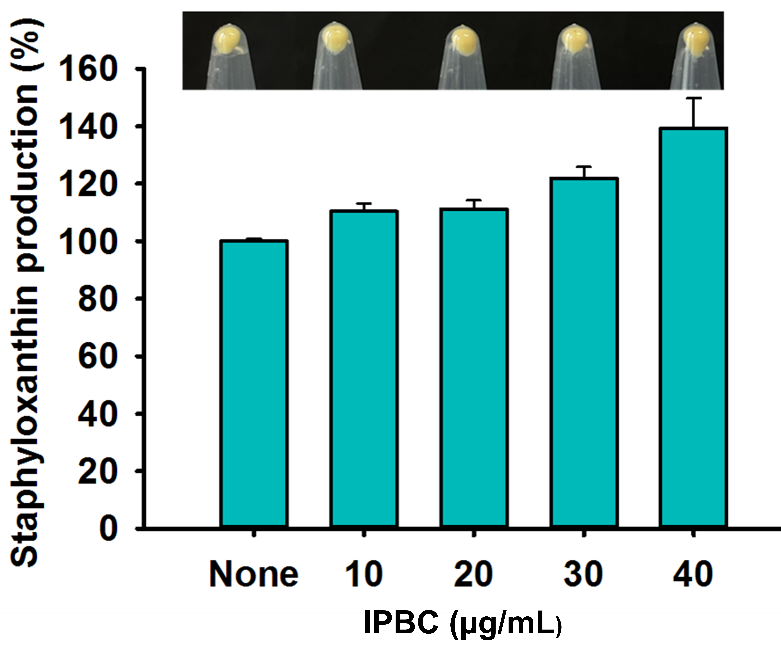


**Supplementary Figure 2.** The effects of IPBC on the staphyloxanthin production in *S. aureus*.


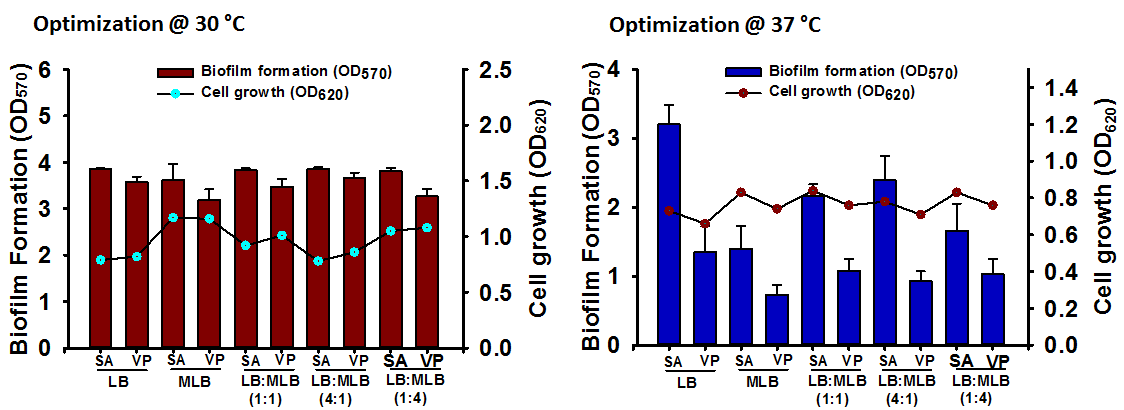


**Supplementary Figure 3.** The optimization for the dual species biofilm formation involving *V. parahaemolyticus* (VP) and *S. aureus* (SA) in different proportions of LB and MLB media at 30 ℃ or 37 ℃.
